# Improving Trainee Doctor Participation in Undergraduate Psychiatry Teaching: A Quality Improvement Project

**DOI:** 10.1192/bjo.2022.304

**Published:** 2022-06-20

**Authors:** Natalie Hughes, Frank Reakes, Jo Davies, Richard Brown, Prakash Muthu

**Affiliations:** 1 Avon and Wiltshire Mental Health Partnership Trust, Bristol, United Kingdom; 2 Gloucestershire Health & Care NHS Foundation Trust, Gloucester, United Kingdom

## Abstract

**Aims:**

Good medical practice encompasses teaching students which is a core competency for trainee doctors. The aim of this project was to assess and improve junior doctor participation in undergraduate psychiatry teaching.

**Methods:**

2 surveys were conducted: 1) Psychiatry-related trainee doctors working in Severn Deanery were emailed a questionnaire to assess their involvement in undergraduate teaching, including barriers and motivators for teaching; 2) doctors with a formal role in teaching were sent a questionnaire to explore their views on recruiting trainee doctors to teach. Questionnaires consisted of multiple answer questions, matrix questions and qualitative free text answer questions. Trainees were then delivered a presentation advertising teaching opportunities. The impact of this on recruitment into psychiatry undergraduate teaching was reassessed by questionnaire.

**Results:**

44 responses were received to the first survey; 13 to the second. The most common answer trainees gave for factors that prevented involvement with teaching students was “unaware of teaching opportunities,” and “lack of overall availability due to clinical commitments.” The most common factor chosen as a motivator for involvement was “notification of session date/timing early in placement” and “protected teaching time in job-plan.” The results highlighted difficulties recruiting trainee doctors to teach, resulting in tutors reducing, cancelling or adapting sessions due to lack of support.

**Conclusion:**

This project identifies barriers and motivators of trainee doctor involvement in undergraduate medical education. To ensure lasting participation of trainees in medical education, support is needed for protected time to teach in clinical roles.

